# Predictors of positive radial margin status in a population-based cohort of patients with rectal cancer

**DOI:** 10.3747/co.v15i2.245

**Published:** 2008-04

**Authors:** P.T. Phang, H. Kennecke, C.E. McGahan, J. MacFarlane, G. McGregor, J.H. Hay

**Affiliations:** Fred Saad, md frcs, Professor of Surgery, U of M Chair in Prostate Cancer Research, Université de Montréal, Director of Urologic Oncology, chum, Montreal, Quebec, Canada, Section Editor

**Keywords:** Rectal cancer, predictors of surgical margin status

## Abstract

**Background:**

Surgical margin status is an important predictor of risk of relapse among patients with rectal cancer.

**Methods:**

Patients referred to the British Columbia Cancer Agency for consideration of adjuvant therapy for rectal adenocarcinoma were included. Predictors of margin positivity were determined from uni- and multivariate analysis.

**Results:**

Among 340 patients, 83% had negative resection margins. In 268 patients with resectable tumours, a significantly higher rate of margin positivity was observed in low rectal tumours (32.2%) as compared with mid-rectal (3.9%) and high rectal (14.3%) tumours. Among 59 patients with locally advanced rectal cancer treated with preoperative radiation (with or without chemotherapy), 32% with low tumours had margin positivity. Of patients with T4 tumours, 50% (11/22) had a positive resection margin.

**Conclusions:**

In a population cohort, distal-third rectal location, locally advanced presentation, and T4 cancer represent subgroups for whom further improvement in therapy is required.

## 1. INTRODUCTION

Previously, outcomes for rectal cancer management in British Columbia were reported for the year 1996^1^. In that retrospective review, worse outcomes were associated with positive margins and a shorter distance from tumour to anus. At that time, BC Cancer Agency guidelines for rectal cancer specified that postoperative chemoradiation be given for stage ii and iii cancers and that preoperative chemoradiation be given for clinically fixed tumours. The 1996 data indicated that the surgical technique of total mesorectal excision (tme) was not consistently performed as the surgical technique of rectal cancer excision.

Subsequently in British Columbia, resectable rectal cancers were treated using preoperative short-course radiation (25 Gy given in 5 daily fractions within 1 week before surgery) with tme as the surgical technique, based on the excellent rectal cancer management outcomes in a Dutch trial^2^. In the present report, we examine radial margins for a provincial population in the year subsequent to that change in management protocol, with an analysis of factors predictive of positive surgical resection margins.

## 2. METHODS

The Colorectal Cancer Outcomes Unit (crcou) database was used to identify all patients with adenocarcinoma of the rectum referred to the BC Cancer Agency from October 1, 2003, to September 30, 2004. The crcou prospectively collects demographic, pathologic, and treatment data for all referred patients with colorectal cancer. Locoregional and distant recurrence and survival are collected prospectively. According to the provincial cancer registry database, 75% of all patients with rectal cancer were referred to the BC Cancer Agency during the period studied. Patients were excluded if they had *in situ* disease, if they had metastatic disease at presentation, if they did not undergo a surgical resection, or if they underwent a local excision only.

Provincial cancer management guidelines in British Columbia were revised to recommend short-course preoperative radiation for all resectable stage ii and iii rectal cancers. Long-course preoperative radiation (≥45) Gy given over 4 or more weeks) in combination with 5-fluorouracil (5-fu) chemotherapy was reserved for locally advanced disease (clinical fixation, or tumour or lymph nodes approaching the predicted mesorectal resection margin), or to increase chances of a sphincter-sparing resection. Postoperative long-course chemoradiation was recommended for stage ii and iii when not given preoperatively. Bolus adjuvant chemotherapy with 5-fu and leucovorin was recommended postoperatively for all patients with stage ii and iii tumours.

Patients were categorized as having resectable cancer or locally advanced cancer, defined clinically as limited mobility or fixed tumour, or radiologically as primary tumour or nodes at or close to mesorectal fascia to the extent that it was unlikely that the tumour could be resected with clear margins. Tumours were also classified by location in the upper (11–15 cm), mid- (6–10 cm), or distal rectum (1–5 cm) according to tumour distance from the anus, and by surgical procedure: anterior resection (antr) or abdominoperineal resection (apr).

The tme specimen grade was assigned either as “complete,” with a grading of “good” (mesorectal fascia intact) or “fair” (minor defects in mesorectal fascia), or as “incomplete” (large defects in mesorectal fascia exposing muscularis of the rectal wall)^3^. A negative margin was recorded if the distance from tumour to the circumferential margin was more than 1 mm.

Univariate and multivariate analyses were used to determine variables predictive of margin positivity and were conducted separately in patients who had resectable and locally advanced rectal cancer. Univariate analysis used the chi-square and Fisher exact tests; patients with unknown values were excluded from the analyses. A 10% significance level was used as the cutoff to enter variables into the multivariate analysis. Multivariate analysis used logistic regression and included only patients with known values for all variables evaluated.

The study received approval from the ubc Research Ethics Board.

## 3. RESULTS

Table I summarizes patient and treatment characteristics for the 340 patients that met the eligibility criteria. Pathologic cancer stage distribution (including downstaging as a result of long-course preoperative treatment) was 2.1% stage 0, 23% stage i, 28% stage ii, 46% stage iii, and 0.9% unknown stage. Tumour location distribution was 29% distal rectum, 54% mid-rectum, and 18% upper rectum.

In 78% of cases, tme was the reported surgical procedure. Quality of tme was reported in only 31% of cases. The tme specimen grade was “complete” in 88% of cases (71% “good,” 17% “fair”) and “incomplete” in 12%^3^. Median number of nodes reported was 11, with 37% of reports including more than 12 nodes, 29% reporting 9–12 nodes, and 31% reporting fewer than 9 nodes. Circumferential margin status was reported in 98% of cases.

A negative margin was achieved in 83% of cases. Table II shows margin positivity according to tumour location and clinical T stage for the resectable group; Table III provides the same information for the locally advanced group. Patients with resectable tumours treated with preoperative short-course radiation or no preoperative radiation had a 12% overall rate of margin positivity as compared with a 32% overall rate of margin positivity for locally advanced tumours that received preoperative long-course radiation. Margin positivity was highest for the distal-third rectal location both in cases that were resectable (32%) and in locally advanced cases (41%). In resectable cases, margin positivity was the lowest for the mid-rectal location (4%); the upper-third rectal location had a 14% rate of margin positivity. Stage T4 tumours partly account for the higher positive margin rate for the upper-third location. Positive margins increased with increasing clinical T stage in resectable and in locally advanced tumours. For resectable tumours, rates of margin positivity were 0% for T1, 8% for T2, 11% for T3. In locally advanced tumours, rates of margin positivity were 22% for T3 and 50% for T4.

The surgical procedure was antr in 63% of cases and apr in 37%. Table IV gives margin status according to surgical procedure and tumour height and describes the four treatment groups. In resectable cases with and without preoperative short-course radiation, positive margin rates in the distal-third rectal location were 11% for antr and 36% for apr. In the mid-rectal location, rates of margin positivity for resectable cases were 5% for antr and 0% for apr. In locally advanced cases that received preoperative long-course radiation, overall rates of margin positivity were 35% for antr and 31% for apr.

Of resectable tumours, overall rates of margin positivity were 11% for preoperative short-course radiation and 16% for no preoperative radiation, with slightly higher rates in cases with no preoperative radiation at all respective tumour locations and clinical T stages (data not shown). A small subgroup of 9 patients with resectable cancers who received preoperative long-course downstaging radiation had a 10% rate of margin positivity.

Table V presents uni- and multivariate analyses of tumour and treatment factors associated with margin positivity in 268 patients with resectable tumours. On univariate analysis, distal rectal location, advanced pathologic and clinical N stage, presence of lymphovascular invasion, and apr were associated with margin positivity. On multivariate analysis, only distal rectal location and advanced pathologic T and N stage were predictive of margin positivity.

Table VI presents univariate analyses of tumour and treatment factors associated with margin positivity in 59 patients with locally advanced tumours that received preoperative long-course radiation. Advanced clinical T stage and pathologic T and N stage and “well” or “moderate” tumour grade were predictive of margin positivity. Mid- and distal rectal locations had equivalent rates of margin positivity in locally advanced tumours. A multivariable analysis could not be carried out because of the small subgroup size.

### 3.1 Exclusions

The study analyses excluded 2 patients with pathologic T0 tumours, 2 patients with pathologic TX tumours, and 1 patient with pathologic NX nodes.

### 3.2 Statistical Analyses

Patients with unknown values were excluded from the univariate analysis, although patients with clinical TX, clinical NX, and unknown lymphovascular invasion were included, because of the large numbers of cases in those categories. The multivariate analyses included 179 patients with known values for all 5 variables.

## 4. DISCUSSION

The present study reviews the effect of a revised provincial protocol on surgical resection margins and assesses factors that predict positive surgical resection margins for rectal cancer. The change in provincial guidelines for rectal cancer management was indicated after a review in 1996 of outcomes for rectal cancer management that showed relatively high rates of pelvic recurrence, particularly for stage iii cancers (27%). In that review, pathology assessment was incomplete, in that only 50% of cases reported radial margin status. Here, we are pleased to find a 98% rate of radial margin assessment. (Although we cannot compare the rate of surgical radial margin negativity in 1996 to the rate in patients treated after the change in the provincial guidelines, we discuss the current overall 83% rate of margin negativity in the context of other agencies using similar management guidelines.) Higher rates of margin positivity were found in the distal-third rectal location and in locally advanced cancer.

For resectable tumours, negative margin rates were 100% for T1, 92% for T2, and 89% for T3. A “complete” tme rate of 88% was reported in a subset of cases. These data likely indicate that surgeons in British Columbia are performing tme as the surgical procedure for rectal cancer excision. By comparison, the Dutch tme study reported a 76% tme “complete” rate^4^. To our knowledge, ours is the first North American report of a population-based outcome for rectal cancer management using the tme protocol.

In patients with resectable rectal cancer, higher margin positivity was observed in distal-third rectal tumours (32.2%) as compared with mid- (3.9%) and upper-third (14.3%) rectal tumours; that higher margin positivity remained significant in the univariate and multivariate analyses alike. Most of the positive margins in upper-third tumours related to anteriorly located tumours that came within 1 mm of, or that perforated, the serosa. These margin positivity rates are similar to those reported in the Dutch tme study: 27% for distal third, 13% for middle third, and 14% for upper third of the rectum^4^.

More-advanced pathologic T and N stage were also independent predictors of margin positivity. A higher rate of margin positivity (32%) was also seen in locally advanced tumours despite preoperative long-course chemoradiation. The highest positive margin rate (50%) occurred in T4 cancers. These data indicate that surgical techniques for clearance of the radial margin for distal-third rectal location and locally advanced tumours may require more attention—with wider, more radical resection—similar to observations by others^5^,^6^.

The tme surgical technique in the distal pelvis is difficult because the confines of the bony pelvis and urogenital organs preclude good visualization of dissection planes. Higher rates of margin positivity occurred for both apr (35%) and antr (31%) of resectable tumours in the distal-third location. These data may indicate that a perineal approach, as in apr, does not provide improved visualization of the dissection planes in the distal third of the rectum. There is no proven technique that will improve visualization and radial margin clearance for the distal-third rectal tumour location, although definitive data are pending for laparoscopic tme and trans-sacral approaches. Furthermore, preoperative assessment of clear margins for cancers in the anterior rectal wall is limited by current imaging modalities. As a result, a decision for *en bloc* resection of anterior urogenital organs in the setting of anterior rectal location must be made on clinical impression.

In addition to improved surgical technique, the Dutch group has suggested increased use of preoperative chemoradiation in an attempt to reduce the rate of margin positivity^5^,^6^. A multicentre Polish rectal cancer trial^7^,^8^ compared short-course preoperative radiation with long-course preoperative chemoradiation in patients with rectal carcinoma in whom at least the inferior margin of the tumour was palpable. In that trial, the rate of margin positivity was significantly lower after preoperative chemoradiation than after short-course treatment (4% compared with 13%)^7^, but the 4-year actuarial local recurrence rates were not significantly different (10.6% after short-course treatment and 14.2% after chemoradiation) 8. The authors rightly point out that surgery follows too soon after short-course treatment to allow for any significant tumour regression; they suggest that if a greater interval is left between radiation and surgery, the rate of margin positivity should fall. That hypothesis is addressed by the ongoing Stockholm iii trial. On the other hand, the overall number of patients with lower-third rectal tumours in the Polish trial was relatively small—a total of 312 patients were analyzed, and the mean inferior distance of the tumour from the anal verge was 5.8 cm^7^. The number of patients in the subgroup may therefore have been too small to detect a difference in local recurrence.

Currently, potentially more-effective preoperative downstaging therapies, including agents such as oxaliplatin and bevacizumab in addition to conventional 5-fu and radiation, are being evaluated in phase ii and iii clinical trials. Patients with distal-third and locally advanced rectal cancers should be referred to those clinical trials. Meanwhile, provincial practice guidelines in British Columbia were amended in 2006 to recommend that all patients with stage ii or iii distal-third rectal cancers be offered preoperative chemoradiation to achieve downstaging.

In the present study, a standard definition of margin positivity was used and defined as tumour within 1 mm or less from the margin. Not all tumours with a close pathologic margin will relapse, but involved margin status has been shown to be associated with an increased risk of both local and distant recurrence, and approximately 30%–40% of patients with positive margins will experience a local or distant event^1^,^9^,^10^. Preoperative radiation (as compared with postoperative radiation) has been associated with improved rates of local recurrence^11^,^12^. Locoregional and distant recurrence will be reported for that cohort once adequate follow-up has been reached.

## 5. CONCLUSIONS

Provincial guidelines for rectal cancer management that include preoperative radiation and tme as the surgical technique for rectal cancer excision have resulted in surgical outcomes similar to those achieved by others using that protocol. Distal-third rectal location and locally advanced cancers had high rates of margin positivity. Provincial practice guidelines have been amended to specify that all patients with stage ii and iii distal-third rectal cancer be treated with preoperative downstaging chemoradiation. To improve surgical outcomes, improved locoregional therapy is urgently required for patients with distal-third rectal cancer.

## Figures and Tables

**TABLE I:** Characteristics and treatment of 340 patients with stages i–iii rectal cancer referred to the British Columbia Cancer Agency, 2003–2004

Patients (*n*)	340
Age (years)	
Median	67
Range	(32–89)
Sex [*n* (%)]	
Men	211 (62)
Women	129 (38)
Surgical procedure [*n* (%)]	
Anterior resection	214 (63)
Abdominoperineal resection	126 (37)
Total mesorectal excision [*n* (%)]	
Done	265 (78)
Not done	61 (18)
Unknown	15 (4.4)
Radiation [*n* (%)]	
Preoperative, short course	181 (53)
Preoperative, long course	68 (20)
Postoperative	47 (14)
None	44 (13)
Chemotherapy [*n* (%)]	
Preoperative	11 (3.2)
Postoperative	119 (35)
Pre- and postoperative	35 (10)
None	175 (51)

**TABLE II:** Margin positivity according to tumour distance from the anus and clinical T stage in 268 patients with resectable tumours who received either no preoperative treatment or short-course radiation

Tumour distance	Cases with positive resection margin [n/N (%)]					
	T1	T2	T3	T4	TX	All
<5 cm	0/3	4/17	11/33	0	4/6	19/59 (32)
5–10 cm	0/5	2/49	2/78	0/3	1/18	5/153 (4)
11–15 cm	0/1	0/8	2/20	0	6/27	8/56 (14)
Total	0/9 (0)	6/74 (8)	15/131 (11)	0/4 (0)	11/51 (22)	32/268 (12)

**TABLE III:** Margin positivity according to tumour distance from the anus and clinical T stage in 59 patients with locally advanced tumours who received preoperative radiation ≥45 Gy, with or without chemotherapy

Tumour distance	Cases with positive resection margin [n/N (%)]			
	T2	T3	T4	All
<5 cm	1/0	6/17	8/17	14/34 (41)
5–10 cm	0/1	2/16	3/5	5/22 (23)
11–15 cm	0/1	0/3	0/0	0/3 (0)
Total	0/1 (1)	8/36 (22)	11/22 (50)	19/59 (32)

**TABLE IV:** Margin status according to surgical procedure and tumour distance from the anus, by treatment group

Treatment group	Surgical procedure	Tumour distance from the anus								
		≤5 cm			6–10 cm			11–15 cm		
		R0	R+		R0	R+	R?	R0	R+	R?
Resectable tumour, pre-op rt 25 Gy in 1 week (*n=*181)	antr	5	1		89	4	1	14	1	1
	apr	26	13		26	0	0	0	0	0
		
		≤*5 cm*			*5–10 cm*			*11–15 cm*		
		*R0*	*R+*		*R0*	*R+*	*R?*	*R0*	*R+*	
		
Resectable tumour, pre-op rt ≥45 Gy, ±chemotherapy^a^ (*n=*9)	antr	1	0		4	0	1	1	0	
	apr	1	0		1	0	0	0	0	
		
		≤*5 cm*			*5–10 cm*			*11–15 cm*		
		
		*R0*	*R+*		*R0*	*R+*		*R0*	*R+*	
Locally advanced tumours,^b^ pre-op rt ≥45 Gy, ±chemotherapy (*n=*59)	antr	2	2		8	4		1	0	
	apr	18	12		9	1		2	0	
		
		≤*5 cm*			*5–10 cm*			*11–15 cm*		
		*R0*	*R+*	*R?*	*R0*	*R+*		*R0*	*R+*	
		
No pre-op treatment (*n=*91)	antr	3	0	0	30	1		33	7	
	apr	6	5		2	3	0	1	0	

a Resectable tumour treated to reduce bulk before attempted anterior resection.

b Defined clinically as limited mobility or fixed tumour, or radiologically as primary tumour or nodes at or close to mesorectal fascia to the extent that tumour resection with clear margins would be an unlikely possibility.

R0 = all margins clear; R+ = margin ≤1 mm or macroscopic residual disease; R? = margin status not specified in pathology report; rt = radiation therapy; antr = anterior resection; apr = abdominoperineal resection.

**TABLE V:** Uni- and multivariate analysis of tumour and treatment factors predictive of positive margin status among 268 patients with resectable tumours treated with short-course preoperative radiation

Tumour and treatment factors	Patients (n)	Proportion with positive margins	Univariate analysis p value (test type)	Multivariate analysis p value (n=179)
Tumour distance from the anus	268		<0.0001 (chi-square)	<0.0019
<5 cm	59	32.2		
5–10 cm	153	3.9		
11–15 cm	56	14.3		
Lymphovascular invasion	182		0.0004 (chi-square)	0.64
Yes	49	26.5		
No	133	6.0		
Surgical procedure	268	8.0	0.0009 (chi-square)	0.87
antr	188	22.5		
apr	80	22.5		
Clinical T stage	268		0.11 (Fisher exact)	
T1	9	0.0		
T2	74	8.1		
T3	131	11.5		
T4	3	0.0		
TX	51	23.5		
Clinical N stage	268		0.0034 (chi-square)	
N0	117	6.8		
N1	52	7.7		
N2	12	8.3		
NX	87	23.0		
Pathologic T stage	264		<0.0001 (chi-square)	<0.0001
T1	17	5.9		
T2	71	1.4		
T3	163	12.9		
T4	13	76.9		
Pathologic N stage	266		<0.0001 (chi-square)	0.02
N0	140	5.0		
N1	79	15.2		
N2	47	29.8		
Tumour grade	262		0.22 (Fisher exact)	
Poor	15	0.0		
Well/moderate	247	13.0		
tme	255		0.78 (chi-square)	
Yes	207	12.5		
No	48	11.1		
Sex	268			
Men	166	11.5	0.58	
Women	102	13.7		

antr = anterior resection; apr = abdominoperineal resection; tme = total mesorectal excision.

**TABLE VI:** Univariate analysis^a^ of tumour and treatment factors predictive of positive margin status among 59 patients who received preoperative radiation with or without chemotherapy

Tumour and treatment factors	Patients (n)	Proportion with positive margins	p Value (test type)
Tumour distance from the anus	59		0.0999 (chi-square)
<5 cm	34	32.2	
≥5 cm	25	20.0	
Lymphovascular invasion	41		0.3061 (chi-square)
Yes	15	46.7	
No	26	26.9	
Surgical procedure	59		0.7659 (chi-square)
antr	17	35.3	
apr	42	31.0	
Clinical T stage	59		0.0423 (Fisher exact)
T1	0		
T2/T3	37	21.6	
T4	22	50.0	
TX	0		
Clinical N stage	59		0.4610 (chi-square)
N0	19	31.6	
N1	24	33.3	
N2	11	18.2	
NX	5	60.0	
Pathologic T stage	56		0.0011 (Fisher exact)
T1	0		
T2	12	0.0	
T3	34	35.3	
T4	10	70.0	
Pathologic N stage	59		0.0159 (chi-square)
N0	34	17.7	
N1	18	50.0	
N2	7	57.1	
Tumour grade	52		0.0395 (Fisher exact)
Poor	8	0.0	
Well/moderate	44	40.9	
tme	57		0.4893 (chi-square)
Yes	45	28.9	
No	12	41.7	

a Patients with unknown values were excluded from the univariate analysis.

antr = anterior resection; apr = abdominoperineal resection; tme = total mesorectal excision.
